# Regional brain atrophy in patients with chronic ankle instability: A voxel-based morphometry study

**DOI:** 10.3389/fnins.2022.984841

**Published:** 2022-09-15

**Authors:** Hui-Min Xie, Zhen-Tong Xing, Zhi-Ye Chen, Xiao-Tan Zhang, Xiao-Juan Qiu, Zi-Shan Jia, Li-Ning Zhang, Xin-Guang Yu

**Affiliations:** ^1^Medical School of Chinese PLA, Beijing, China; ^2^Department of Rehabilitation Medicine, The First Medical Centre, Chinese PLA General Hospital, Beijing, China; ^3^Department of Rehabilitation Medicine, Hainan Hospital of Chinese PLA General Hospital, Sanya, China; ^4^Department of Radiology, Hainan Hospital of Chinese PLA General Hospital, Sanya, China; ^5^Department of Neurosurgery, Chinese PLA General Hospital, Beijing, China

**Keywords:** ankle instability, ankle sprain, brain atrophy, brain region, gray matter volume, neural plasticity, proprioception, visual analog scale

## Abstract

The objective of this study was to investigate whether brain volume changes occur in patients with chronic ankle instability (CAI) using voxel-based morphometry and assessing correlations with clinical tests. Structural magnetic resonance imaging data were prospectively acquired in 24 patients with CAI and 34 healthy controls. CAI symptoms and pain intensity were assessed using the Foot and Ankle Ability Measure (FAAM), Cumberland Ankle Instability Tool (CAIT), American Orthopedic Foot and Ankle Society (AOFAS) ankle-hindfoot score, and visual analog scale (VAS). The gray matter volume (GMV) of each voxel was compared between the two groups while controlling for age, sex, weight, and education level. Correlation analysis was performed to identify associations between abnormal GMV regions and the FAAM score, AOFAS score, VAS score, disease duration, and body mass index. Patients with CAI exhibited reduced GMV in the right precentral and postcentral areas, right parahippocampal area, left thalamus, left parahippocampal area, and left postcentral area compared to that of healthy controls. Furthermore, the right parahippocampal (r = 0.642, *p* = 0.001), left parahippocampal (r = 0.486, *p* = 0.016), and left postcentral areas (r = 0.521, *p* = 0.009) were positively correlated with disease duration. The left thalamus was positively correlated with the CAIT score and FAAM activities of daily living score (r = 0.463, *p* = 0.023 and r = 0.561, *p* = 0.004, respectively). A significant positive correlation was found between the local GMV of the right and left parahippocampal areas (r = 0.487, *p* = 0.016 and r = 0.763, *p* < 0.001, respectively) and the AOFAS score. Neural plasticity may occur in the precentral and postcentral areas, parahippocampal area, and thalamus in patients with CAI. The patterns of structural reorganization in patients with CAI may provide useful information on the neuropathological mechanisms of CAI.

## Introduction

Ankle sprains, a common injury in daily life, are not a one-time injury. A common sequela is the development of chronic ankle instability (CAI), a condition characterized by recurrent sprains and/or repetitive “giving way” of the ankle (Ardakani et al., 2019). Approximately 32% to 74% of individuals with a history of ankle sprain suffer from residual and chronic symptoms, recurrent ankle sprains, and/or perceived instability (Gribble et al., 2014). CAI has previously been divided into mechanical ankle instability and functional ankle instability (Hertel, 2002). However, the International Ankle Consortium has provided a succinct definition of CAI that focuses on repetitive episodes of giving away and self-reported dysfunction. This literature has minimized the difference in the definitions of functional or mechanical ankle instability (Gribble et al., 2014) and emphasized the complex factors that contribute to CAI (Gribble, 2019). CAI is often accompanied by pain, instability, and abnormal ankle activity. Chronic stimulation (such as pain or amputation) is known to cause cortical neuroplasticity, which results in numerous sensory or functional changes. CAI was previously demonstrated to negatively alter central mechanisms of motor control (Doherty et al., 2014). Wikstrom et al. (2010) reported that patients with CAI showed alterations in feed-forward neuromuscular control using surface electromyography and suggested the presence of feedback neuromuscular control deficits. Hass et al. (2010) also reported that supraspinal motor control mechanisms were altered in patients with CAI, emphasizing the importance of minimizing postural demands on the involved limb. Thus, this study explored whether neural remodeling would occur in the cerebral cortex of patients with CAI.

Voxel-based morphometry (VBM) has been widely used to quantitatively study the location and degree of structural changes in the brain associated with development, aging, and degeneration (Ashburner et al., 2003). VBM has been recently used to evaluate central neural plasticity caused by peripheral and chronic diseases, such as acute eye pain, persistent pain following total knee arthroplasty, and painful temporomandibular disorder (Lan et al., 2019; Chen et al., 2020; Lewis et al., 2020). These findings may reveal the relationship between the structural and functional adaptations of the human body. In Lewis et al.'s (2020) article, 45 patients with persistent pain following total knee arthroplasty were examined and analyzed using VBM. The gray matter density of the right amygdala, right nucleus accumbens, and periaqueductal gray correlated positively with temporal summation of pain. However, a recent study revealed that chronic musculoskeletal impairment was associated with alterations in brain regions responsible for the production and perception of movement (Conboy et al., 2021). In this study, 21 patients with massive irreparable rotator cuff tear and 13 healthy controls were enrolled to study the impact of chronic motor impairment on the human brain. Lower gray matter density and cortical thickness were observed in the postcentral gyrus, inferior parietal lobule, temporal-parietal junction, and pulvinar areas implicated in somatosensation, reach/grasp, and body form perception.

To the best of our knowledge, this study is the first to report regional brain atrophy in patients with CAI. Xue et al. (2022) made an initial attempt to study the corticospinal tract using diffusion tensor imaging analysis, and their results revealed that the contralateral corticospinal tract of the unstable ankle in patients with CAI exhibited impaired integrity. Resting-state functional magnetic resonance imaging (MRI) was used by Shen et al. (2022), who found that patients with CAI exhibited more enhanced and coherent regional inherent neuronal activity within the sensorimotor network. In our study, the aim was to investigate the relationship between brain volume changes and CAI using VBM-based gray matter (GM) volume (GMV) to visualize brain alterations associated with ankle function and local pain. We hypothesized that long-term dyskinesia and pain may cause changes in the GMV, which in turn would cause neural remodeling.

## Materials and methods

### Participants

All patients and healthy controls (HCs) were recruited from January 2019 to December 2021. The participants were male and aged between 20 and 45 years. CAI-specific criteria were consistent with the recommendations of the International Ankle Consortium (Gribble et al., 2014). The inclusion criteria for the intervention group were as follows: (1) initial sprain occurred at least 12 months prior to study enrollment; (2) at least one acute ankle-inversion sprain that resulted in swelling, pain, and dysfunction; (3) most recent injury occurred more than 3 months prior to study enrollment; (4) at least two episodes of the ankle “giving way” within the previous 6 months; (5) Cumberland Ankle Instability Tool (CAIT) score < 24 (Hiller et al., 2006); (6) Foot and Ankle Ability Measure (FAAM)-activities of daily living (ADL) scale < 90% and sport scale < 80% (Carcia et al., 2008); and (7) absence of other chronic, intracranial, or psychological diseases. The exclusion criteria for the intervention group were as follows: (1) a history of previous surgery on musculoskeletal structures; (2) acute injury to the musculoskeletal structures of other joints in the lower extremity in the previous 3 months; (3) presence of other chronic, intracranial, or psychological diseases; and (4) MRI could not be performed due to any reason (metal, pacemaker, confined space phobia, etc.). The inclusion criteria for the HC group were: (1) absence of any chronic, intracranial, or psychological diseases; and (2) no history of ankle sprain. The studies involving human participants were reviewed and approved by the Research Ethical Board of the Chinese PLA General Hospital (No.S2021-148-01). The patients/participants provided their written informed consent to participate in this study.

### Clinical information

Patient data, including body mass index (BMI), age, disease duration, diagnosis, and the visual analog scale (VAS), FAAM-ADL, FAAM-sports, CAIT, and American Orthopedic Foot and Ankle Society (AOFAS) ankle-hindfoot scores, were collected.

### MRI acquisition

Serial MRI scans were obtained on a 3.0-T Skyra MRI scanner (Discovery MR750, GE Healthcare, Chicago, IL, USA). The axial three-dimensional T1-weighted fast spoiled gradient-recalled echo sequence was performed for all participants using the following imaging parameters: repetition time = 6.3 ms, echo time = 2.8 ms, flip angle = 15°, field of view = 25.6 × 25.6 cm, matrix = 256 × 256, number of acquisitions = 1, and slice thickness = 1 mm.

### VBM analyses

Three-dimensional T1-weighted fast spoiled gradient-recalled echo MR images were processed with VBM8 using SPM8 (http://www.fil.ion.ucl.ac.uk/spm/software/spm8/). All three-dimensional T1-weighted MRI scans were normalized using an affine followed by non-linear registration, corrected for bias field in homogeneities, and then segmented into GM, white matter (WM), and cerebrospinal fluid components in the preprocessing steps. The Diffeomorphic Anatomic Registration Trough Exponentiated Lie algebra algorithm (DARTEL) tool was used to generate GM and WM templates and normalize the segmented scans into a standard Montreal Neurological Institute (MNI) space, which were then used to obtain the standardized GM of all participants (Farokhian et al., 2017). The volume was then smoothed with an 8-mm full width at half maximum Gaussian kernel. The normalized, modulated, and smoothed image was used to analyze the group level. Within-group regression analyses were performed using SPM8. After adjusting for age, the differences in GM between patients with CAI and HCs were compared. The level of statistical significance was set at *p* < 0.05, and the Gaussian random field theory correction was used (minimum z > 2.3).

### Statistical analysis

Correlation analysis was used to calculate the relationship between average GM values per voxel of each individual participant from the clusters of interest and clinical data (duration, BMI, VAS, CAIT, and FAAM). A two-sample *t*-test between the two groups was performed to compare age, BMI, and disease duration. Data are presented as the mean ± standard deviation. Statistical significance was set at *p* < 0.05.

## Results

### Clinical characteristics of the sample

The study included 24 male participants with CAI and 34 male HCs from January 2019 to December 2021. All patients who met the inclusion criteria were included in the study. There were no significant differences in terms of age (25.54 ± 3.11 years and 25.84 ± 3.15 years, respectively; *p* = 0.72) and BMI (22.72 ± 2.13 kg/m^2^ and 22.45 ± 2.09 kg/m^2^, respectively; *p* = 0.58) between the CAI and HC groups. The CAI group comprised 12 patients with left-sided injuries and 13 patients with right-sided injuries. The mean disease duration was 19.25 ± 9.54 months. The mean VAS, CAIT, FAAM-ADL, FAAM-sports, and AOFAS scores were 4.08 ± 1.96, 17.96 ± 4.22, 57.92 ± 13.2, 21.29 ± 2.88, and 69.71 ± 13.2, respectively (Table 1).

**Table 1:** Clinical characteristics of the subjects.

	**CAI**	**HC**	**T value**	***P* value**
Age	25.54 (3.11)	25.84 (3.15)	0.36	0.72
BMI	22.78 (2.12)	22.45 (2.09)	0.56	0.58
Duration	12.32 (9.33)	NA	NA	NA
Injury side				
Left	12	NA	NA	NA
Right	12	NA	NA	NA
VAS	4.08 (1.96)	NA	NA	NA
CAIT	17.96 (3.69)	NA	NA	NA
FAAM-ADL	59.12 (11.65)	NA	NA	NA
FAAM-SPORT	20.72 (4.25)	NA	NA	NA
AOFAS	70.04 (12.38)	NA	NA	NA

AOFAS, American Orthopedic Foot and Ankle Society; BMI, body mass index; CAI, chronic ankle instability; CAIT, Cumberland Ankle Instability Tool; FAAM, Foot and Ankle Ability Measure; HC, healthy control; NA, not available; VAS, Visual Analog Scale.

### Comparison of brain GMV between the CAI and HC groups

**Figure 1:**
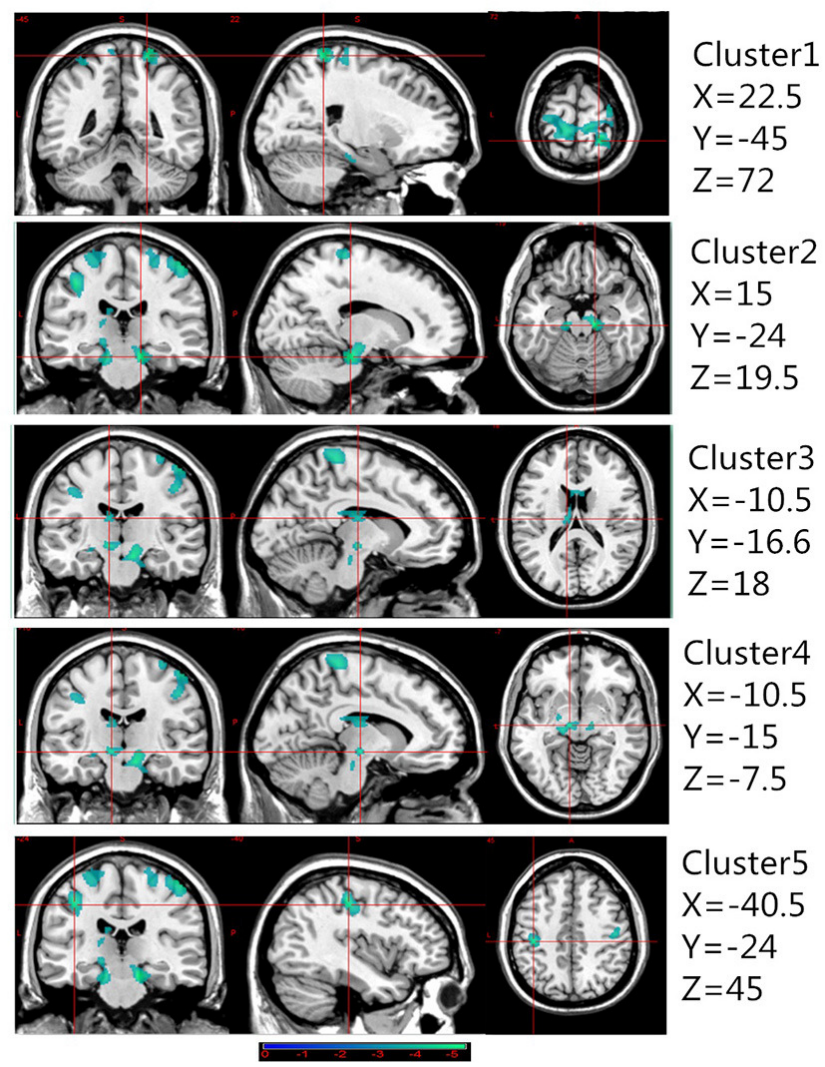
Gray matter volume decreased in specific cerebral areas in the chronic ankle instability (CAI) group compared with the healthy control group. Regions with decreased gray matter (clusters 1 to 5) in the CAI group are superimposed on a normalized structural cerebral image. The color bar represents the t-score. Coordinates (X, Y, and Z values) are given in the Montreal Neurological Institute (MNI) space.

**Figure 2:**
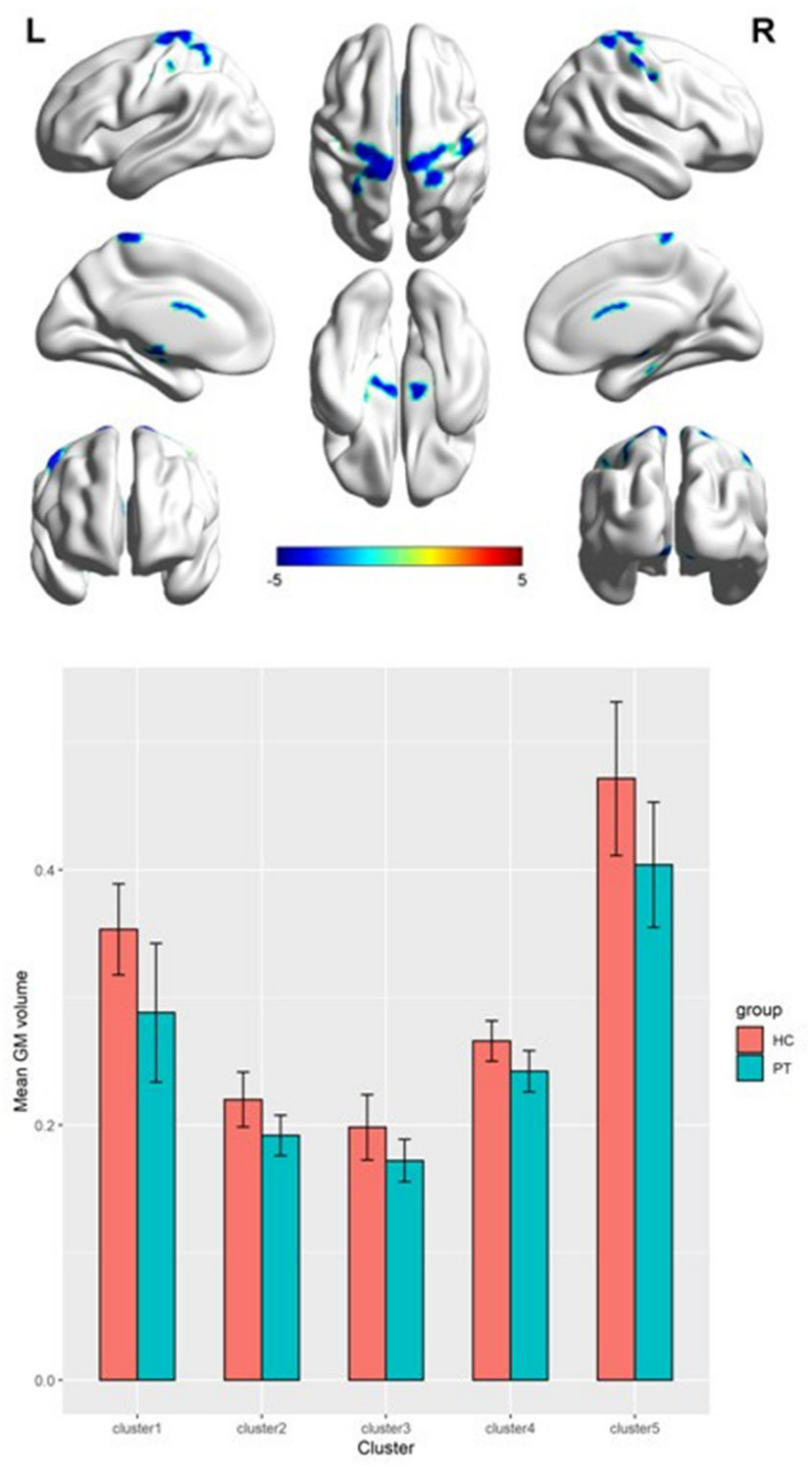
A 3D image of the significant decrease in gray matter volume in the chronic ankle instability (CAI) group. Cluster 1: Montreal Neurological Institute (MNI) coordinate: 22.5, −45, 72; cluster 2: MNI coordinate: 15, −24, −19.5; cluster 3: MNI coordinate: −10.5, −16.5, 18; cluster 4: MNI coordinate: −10.5, −15, −7.5; cluster 5: MNI coordinate: −40.5, −24, 45.

Table 2 presents the following brain regions with volume reduction in patients with CAI compared to those in HCs: right precentral and postcentral areas (cluster 1), right parahippocampal area (cluster 2), left thalamus (cluster 3), left parahippocampal area (cluster 4), and left postcentral area (cluster 5). No brain region showed a volume increase in patients with CAI compared to that of HCs (Figures 1, 2). There were no statistical differences between the CAI patients with left- and right-sided injuries in terms of contralateral or ipsilateral brain volumes. The left and right brain mean GMVs of CAI patients with right-sided injuries were 0.42 ± 0.023 and 0.41 ± 0.021, respectively (*p* = 0.29), while they were 0.413 ± 0.026 and 0.41 ± 0.029, respectively, in patients with left-sided injuries (*p* = 0.25). The left and right brain GMVs between CAI patients and HCs also showed no statistical differences (left mean GMV: 0.421 and 0.412, respectively, *p* = 0.25; right mean GMV: 0.418 and 0.41, respectively, *p* = 0.3).

**Table 2:** Brain gray matter volume with volume gain in patients with chronic ankle instability (CAI) compared to healthy controls.

**Anatomic regions**	**MNI space**			**Cluster size**	**T value**	***P* value**
	**X**	**Y**	**Z**			
Right precentral and postcentral area	22.5	−45	72	4,516	−5.099	0.000
Right parahippocampal area	15	−24	−19.5	869	−5.353	0.000
Left thalamus	−10.5	−16.5	18	804	−3.825	0.000
Left parahippocampal area	−10.5	−15	−7.5	762	−4.638	0.000
Left postcentral area	−40.5	−24	45	726	−4.945	0.000

### Voxel-wise correlation analysis between brain volume and clinical variables

**Figure 3:**
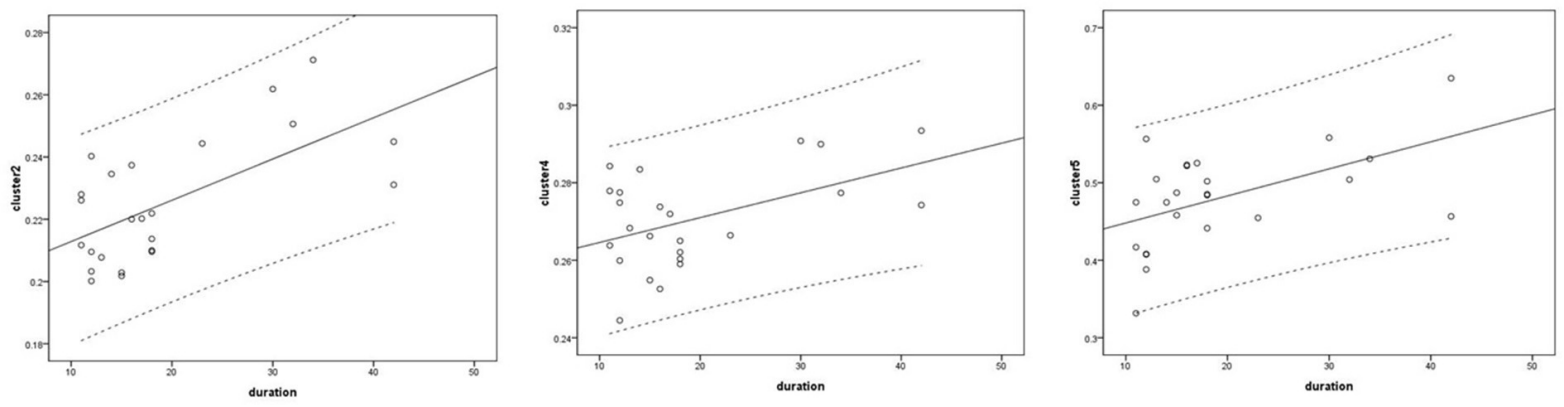
Correlation analysis between clusters and disease duration. Cluster 2: r = 0.642; cluster 4: r = 0.486; cluster 5: r = 0.521.

**Figure 4:**
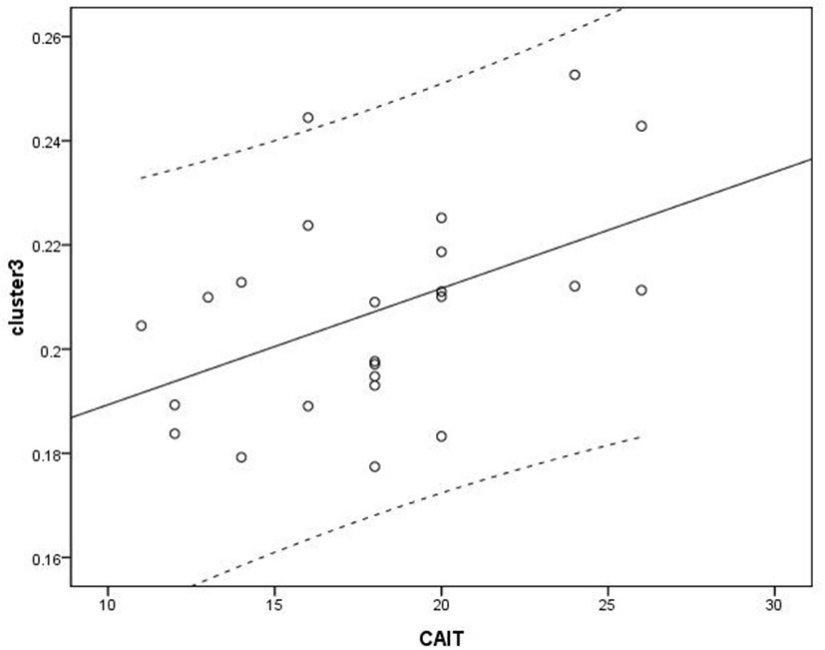
Correlation analysis between cluster 3 and Correlation Cumberland Ankle Instability Tool (CAIT). Cluster 3: r = 0.463.

**Figure 5:**
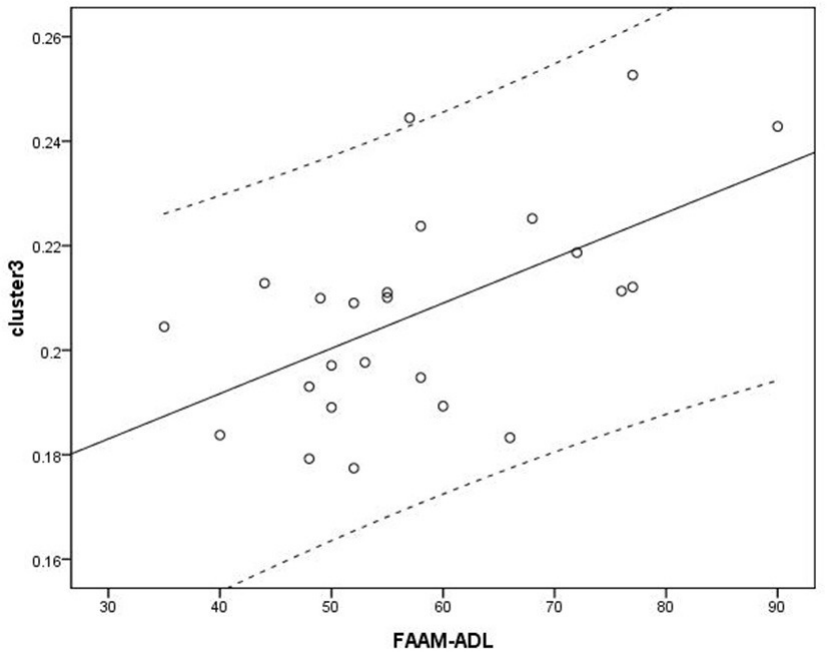
Analysis between cluster 3 and Foot and Ankle Ability Measure activities of daily living (FAAM-ADL) scores. Cluster 3: r = 0.561.

**Figure 6:**
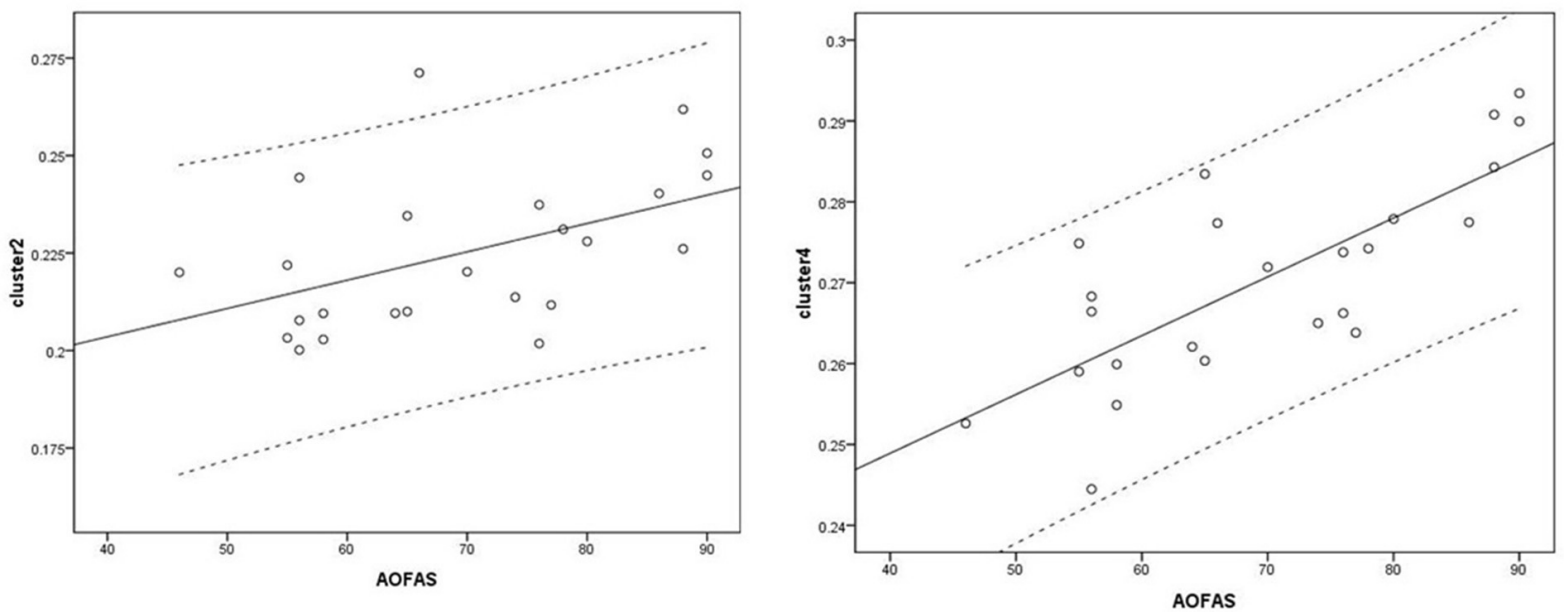
Correlation analysis between clusters and American Orthopedic Foot and Ankle Society (AOFAS) scores. Cluster 2: r = 0.487; Cluster 4: r = 0.763.

Voxel-based correlation analysis demonstrated that the right parahippocampal area (MNI coordinate: 15, −24, −19.5; cluster size: 869; r = 0.642, *p* = 0.001), left parahippocampal area (MNI coordinate: −10.5, −15, −7.5; cluster size: 762; r = 0.486, *p* = 0.016), and left postcentral area (MNI coordinate: −40.5, −24, 45; cluster size: 726; r = 0.521, *p* = 0.009) were positively correlated with disease duration (Figure 3). The left thalamus (MNI coordinate: −10.5, −16.5, 18; cluster size: 804) was positively correlated with the CAIT and FAAM-ADL scores (r = 0.463, *p* = 0.023 and r = 0.561, *p* = 0.004, respectively) (Figures 4, 5). The left parahippocampal area (MNI coordinate: −10.5, −15, −7.5; cluster size: 762; r = 0.763, *p* < 0.001) and the right parahippocampal area (MNI coordinate: 15, −24, −19.5; cluster size: 869; r = 0.487, *p* = 0.016) were positively correlated with AOFAS score (Figure 6). The other clinical variables, including VAS score, BMI, and FAAM-sports score, showed no correlations with brainstem volume.

## Discussion

To the best of our knowledge, this study is the first to report regional brain atrophy in patients with CAI. Compared with HCs, patients with CAI showed five clusters of significant correlations between the right precentral and postcentral areas, right parahippocampal area, left thalamus, left parahippocampal area, and left postcentral area. These results indicate that structural brain plasticity may occur in interconnected areas in patients with CAI. Voxel-wise correlation analysis also revealed that there may be a strong correlation between the left parahippocampal area and AOFAS score (Schober et al., 2018). In addition, there may be moderate correlations between the parahippocampal area and disease duration, the left postcentral area and disease duration, the left thalamus and CAIT score, the left thalamus and FAAM-ADL score, and the right parahippocampal area and AOFAS score.

The results showed that structural brain plasticity is more likely to occur in the parahippocampal and left postcentral areas of patients with CAI who have a long disease duration. The postcentral gyrus is known to contain the primary somatosensory cortex, a brain region responsible for proprioception (DiGuiseppi and Tadi, 2021). A previous study showed that proprioceptive capabilities decreased following joint injuries, such as ankle sprain, and abnormalities may vary with the course of the disease and the extent of the injury (Jerosch and Prymka, 1996). This may be a reasonable explanation for the results of this study. Loprinzi (2019) summarized the possible effects of exercise on the parahippocampal region in 20 human and 8 animal models. The evaluation methods of neural activity included blood oxygen level-dependent signal changes, glucose metabolism, functional connectivity, fractional anisotropy, and brain volume. They concluded that exercise appeared to have a significant effect on parahippocampal function. Patients with CAI may have declined motor capacity due to pain, ankle discomfort, or fear of re-injury. Thus, the reduction in movement causes neural remodeling in the parahippocampal region, which leads to a reduction in the volume of the parahippocampal region. Siddarth et al. (2018a) performed a cross-sectional study on 35 adults and found that higher levels of sitting were associated with lower volume in the parahippocampal cortex. Higher physical activity level was also associated with greater parahippocampal volume in individuals aged > 60 years (Siddarth et al., 2018b). These results are consistent with those obtained in the present study.

The FAAM is a reliable, valid, and responsive self-reported physical scale that can comprehensively assess physical function in individuals with musculoskeletal disorders of the leg, foot, and ankle. It consists of 21-item ADL (FAAM-ADL) and 8-item physical activity (FAAM-sports) subscales (Carcia et al., 2008). The FAAM-ADL subscale includes common actions in daily life, such as standing, walking on even or uneven ground, walking up and down hills, and going up and down stairs. The FAAM-sports subscale includes more difficult movements, such as running, jumping, starting and stopping quickly, and the ability to participate in a patient's desired sport as long as they would like. In the present study, voxel-wise correlation analysis showed that the left thalamus was positively correlated with FAAM-ADL score. The thalamus, which has extensive connections with the cerebral cortex, is known for its relay function and plays an important role in the top-down transmission and regulation of acute and chronic pain (Li et al., 2021). In addition, the thalamus plays a crucial part in motor control and motor learning (Sommer, 2003; Hasegawa et al., 2020). A previous study found that the motor performance of mice improved after exercise training and their thalamic neuronal synaptic plasticity strengthened, indicating that exercise can affect the thalamus (Ding et al., 2002). Furthermore, Jung et al. (2019) performed intermanual transfer training of the non-dominant hand on 20 healthy adults. They found that after the training, neuroplasticity in the thalamus was closely related to motor behavioral change (Jung et al., 2019). The thalamus is associated with motor ability, pattern, and pain, which may explain the results of this study. However, no statistically significant correlation was found between GMV and FAAM-sports score. The possible reason may be the lack of significant difference in FAAM-sports scores (18–24).

The AOFAS ankle-hindfoot score has been widely used as a region-specific health outcome measure to assess the outcomes of patients with foot and ankle injuries. This scale includes three aspects of assessment: pain, function, and alignment (Schneider and Jurenitsch, 2016). In the present study, the parahippocampal area was positively correlated with AOFAS score, which indicated that the decrease in ankle comprehensive function may cause neural remodeling in the parahippocampal region.

Correlation analysis between the VAS and GMV was also performed, but no significant correlation was found. This finding suggests that the neuroplasticity involved in this study may be related to motor control and sensory dysfunction networks rather than pain networks. Research on neuroplasticity in patients with chronic peripheral pain (fibromyalgia, knee arthritis, phantom limb pain [PLP]) or spinal cord injury is a hot topic. However, few studies have investigated the relationship between dyskinesia due to non-neurological injury and cortical neuroplasticity. Currently, cortical remapping theory, which is the brain's response to limb loss by reorganizing somatosensory maps, is a widely accepted theory to explain PLP. Bogdanov et al. (2012) found that movements of the intact hand were accompanied by increased activity in the sensorimotor cortices of both cerebral hemispheres, indicating that the cerebral cortex associated with the amputated limb was non-adaptive to functional reorganization. Draganski et al. (2006) compared the brain structure of 28 patients with unilateral limb amputation. They reported that the thalamic GM differences were positively correlated with the time span after amputation but not with the frequency or magnitude of coexisting phantom pain. Preissler et al. (2013) investigated the GMV of 21 patients with medium-to-high PLP or slight PLP. The results indicated that all patients may have had an enhanced need for visual control to compensate for the lack of sensory feedback of the missing limb. In addition to the extreme case of amputated limbs, some researchers have investigated neural remodeling in the cerebral cortex of neuro-injured patients with dyskinesia. Most of the aforementioned studies focused on changes in the central nervous system after spinal cord injury. Zhang et al. (2015, 2021) used a mouse model of spinal cord hemisection to observe changes in the cerebral cortex due to movement disorders caused by spinal cord injury. Interestingly, the cortical dendritic spines of the bilateral sensory cortex and motor cortex developed and plasticized. In addition, cortical remodeling induced by motor learning was preliminary studied by several groups. Stevenson et al. (2021) found that motor-skill learning was associated with cerebellar synaptogenesis and astrocytic hypertrophy. In the human central nervous system, preliminary evidence shows that long-term physical activity leads to specific functional and structural brain alterations (Seidel et al., 2017). Meier et al. (2016) also found that GMV increased in the hands of handball players and the feet of ballet dancers.

At present, the possible mechanism of CAI includes the following (Hertel, 2002; Sefton et al., 2009; Hiller et al., 2011; Croy et al., 2012; Terada et al., 2017; McCann et al., 2019; Xue et al., 2021): (1) local injury (such as pathological laxity, arthrokinematic restrictions, degenerative changes, and synovial changes); (2) strength deficits (including around the ankle or hip); (3) impaired proprioception; (4) impaired neuromuscular control; and (5) impaired postural control. In recent years, the relation between CAI and changes in the central nervous system processing mechanism has been investigated. Song et al. (2016) believe that patients with CAI upregulate the use of visual information during single-limb stance, which indicates that impaired proprioception may cause the re-weighting of sensory afferent. Witchalls et al. (2014) conducted repeated proprioception sensitivity testing to compare the performances of CAI and stable ankle groups. The test results showed that the proprioception scores of participants with CAI improved more slowly. This suggests that lower learning strategies may occur because of neural remodeling. To compare the corticomotor map output for the fibularis longus (FL) muscle in patients with and without CAI, Kosik et al. (2017) used transcranial magnetic stimulation to map the motor cortex's representation. They concluded that a highly concentrated area of neurons communicated with the FL muscle in patients with CAI. This finding indicates that the motor cortical cells on the border of the FL excitation area are less involved to the proper function of the FL muscle and may be recruited by other surrounding areas. In our study, we discussed the possible mechanisms and neuroplasticity of CAI using VBM analyses. In previous studies, long-term career choice and chronic illness influenced GMV (Lan et al., 2019; Chen et al., 2020; Lewis et al., 2020; Qiu et al., 2021). Moreover, Anderson (2011) summarized the evidence of the relationship between regional brain volume and dendritic length in both animals and humans. Long-term training and chronic illness may increase regional volume by increasing dendritic branching and synapse numbers. Additionally, training and illness may be re-affected by neural plasticity. However, whether neural regulation treatment can ameliorate dysfunction may be a direction of future research.

This study has three main limitations. First, this was a cross-sectional observational study. We will perform a randomized controlled trial in the future to validate our findings. Second, because of the small sample size, subgroup analysis, such as left–right, injuried type and sex differences, was not performed. Third, because of the particularity of the military hospital, all the patients enrolled in this study were male, and the findings of ankle MRI showed obvious mechanical injuries. In this study, preliminary findings showed volume reduction in some brain areas in patients with CAI using VBM. Further studies using other methods, such as functional MRI and diffusion tensor imaging, are also warranted. In addition, it is an interesting question as to whether neuromodulation techniques can be used as an adjunctive therapy for patients with refractory CAI. Our team has started to design experiments to explore this problem.

In conclusion, neural plasticity may occur in the precentral and postcentral areas, the parahippocampal area, and the thalamus in patients with CAI. The patterns of structural reorganization in patients with CAI may provide useful information on the neuropathological mechanisms of CAI.

## Data availability statement

The raw data supporting the conclusions of this article will be made available by the authors, without undue reservation.

## Ethics statement

The studies involving human participants were reviewed and approved by the Research Ethical Board of the Chinese PLA General Hospital (No. S2021-148-01). The patients/participants provided their written informed consent to participate in this study. Written informed consent was obtained from the individual(s) for the publication of any potentially identifiable images or data included in this article.

## Author contributions

H-MX, X-GY, Z-SJ, and L-NZ designed the experiment. Z-YC and Z-TX analyzed the data. X-TZ checked the data. X-JQ assessed the clinical information of the patients. H-MX wrote the draft of the manuscript. All authors discussed the results, commented on the manuscript, and approved the submitted version.

## Funding

The present study was supported by the National Key Research and Development Program of China (No. 2019YFB1311402), the Scientific Research Program of General Logistics Department of PLA (AMS17J004), and the Military Innovative Medicial Program of PLA General Hospital (CX19005).

## Conflict of interest

The authors declare that the research was conducted in the absence of any commercial or financial relationships that could be construed as a potential conflict of interest.

## Publisher's note

All claims expressed in this article are solely those of the authors and do not necessarily represent those of their affiliated organizations, or those of the publisher, the editors and the reviewers. Any product that may be evaluated in this article, or claim that may be made by its manufacturer, is not guaranteed or endorsed by the publisher.
